# Aging Increases Susceptibility to Develop Cardiac Hypertrophy following High Sugar Consumption

**DOI:** 10.3390/nu14214645

**Published:** 2022-11-03

**Authors:** Ana P. Valencia, Jeremy A. Whitson, Shari Wang, Leon Nguyen, Laura J. den Hartigh, Peter S. Rabinovitch, David J. Marcinek

**Affiliations:** 1 Department of Radiology, University of Washington, Seattle, WA 98109, USA; 2 Department of Biology, High Point University, High Point, NC 27268, USA; 3 Department of Medicine, Metabolism, University of Washington, Seattle, WA 98109, USA; 4 Arizona College of Osteopathic Medicine, Midwestern University, Glendale, AZ 85308, USA; 5 Department of Pathology, University of Washington, Seattle, WA 98195, USA

**Keywords:** heart disease, heart failure, mitochondrial dysfunction, diet, inflammation, age-related disease, sucrose, SS-31, fructose

## Abstract

Aging and poor diet are independent risk factors for heart disease, but the impact of high-sucrose (HS) consumption in the aging heart is understudied. Aging leads to impairments in mitochondrial function that result in muscle dysfunction (e.g., cardiac remodeling and sarcopenia). We tested whether HS diet (60%kcal sucrose) would accelerate muscle dysfunction in 24-month-old male CB6F1 mice. By week 1 on HS diet, mice developed significant cardiac hypertrophy compared to age-matched chow-fed controls. The increased weight of the heart persisted throughout the 4-week treatment, while body weight and strength declined more rapidly than controls. We then tested whether HS diet could worsen cardiac dysfunction in old mice and if the mitochondrial-targeted drug, elamipretide (ELAM), could prevent the diet-induced effect. Old and young mice were treated with either ELAM or saline as a control for 2 weeks, and provided with HS diet or chow on the last week. As demonstrated in the previous experiment, old mice had age-related cardiac hypertrophy that worsened after one week on HS and was prevented by ELAM treatment, while the HS diet had no detectable effect on hypertrophy in the young mice. As expected, mitochondrial respiration and reactive oxygen species (ROS) production were altered by age, but were not significantly affected by HS diet or ELAM. Our findings highlight the vulnerability of the aged heart to HS diet that can be prevented by systemic targeting of the mitochondria with ELAM.

## 1. Introduction

Aging and poor nutrition are major risk factors for heart disease, which is currently the leading cause of death in people over the age of 65. Growing epidemiological evidence published within the past decade has shown the negative impact of simple sugar consumption on heart health. According to the *Dietary Guidelines for Americans*, energy from added sugars should not exceed 10% of energy intake, but it is estimated that 71% of the population consumes more than this recommendation. Importantly, Yang et al., reported that cardiovascular risk mortality rises exponentially with increasing levels of sugar consumption, and participants in the highest quintile who consumed >21% of calories from sugar had more than double the risk of cardiovascular disease mortality compared to those consuming less than 8% of calories from sugar [1]. There has been a striking increase in the consumption of simple sugars over the past years as processed foods become mainstream. The major sources contributing to excess sugar consumption are sugar-sweetened beverages, which have also been associated with greater risk for congestive heart failure in African Americans independent of BMI [2]. One of the most widely used sweeteners is table sugar, also known as sucrose. It is a disaccharide made up of a glucose and fructose unit in a 1:1 ratio. High fructose corn syrup is also commonly used and contains a greater proportion of fructose over glucose. Fructose is recognized as a mediator of metabolic diseases, and its consumption continues to increase with the widespread use of these sweeteners. Interestingly, glucose accelerates the rate of fructose disposal into the portal vein, and can also be converted into fructose [3,4]. It is generally recognized that sugar consumption can promote obesity and type 2 diabetes, both of which are significant risk factors for heart disease. However, less is known about the effect of sugar consumption on the aging heart in the absence of weight gain and diabetes.

The aging heart is characterized by disrupted metabolism and mitochondrial dysfunction that can increase susceptibility to nutritional stressors [5]. An important role for mitochondria in the aging heart is supported by recent reports that cardiac hypertrophy and dysfunction in aged rodents can be ameliorated by mitochondrial-targeted therapies, such as elamipretide (ELAM) [6,7]. ELAM is a synthetic tetrapeptide that associates with the head group of cardiolipin, a phospholipid specific to the inner mitochondrial membrane [8]. Left ventricular hypertrophy is a well-recognized risk factor for future adverse cardiac events, with greater evidence of pathological cardiac hypertrophy in old age. Sugar consumption promotes cardiac hypertrophy in young mice [9,10] and exacerbates cardiac pathology in young rodent models of pressure overload [11,12], but to our knowledge there are no published studies examining the effects of sugar consumption in cardiac health in the context of aging. The purpose of this study was to test whether the aging heart is more susceptible to nutritional stress induced by a high-sucrose diet. We tested the involvement of mitochondria in this interaction by determining whether ELAM can prevent this effect. We found that after only one week of high-sucrose (HS) diet, cardiac hypertrophy was accelerated in aged mice, but young mice and aged mice treated with ELAM were protected.

## 2. Materials and Methods

### 2.1. Animal Care and Treatment Groups

This study was approved by the Institutional Animal Care and Use Committee of the University of Washington. All animals were used in accordance with the National Institute of Health guidelines for the care and use of laboratory animals. Male CB6F1 hybrid mice (BALB/cBy × C57BL/6) were obtained from the National Institute of Aging (NIA) resource. All mice were group-housed and exposed to a 14 h light:10 h dark cycle in a fixed-temperature environment with free access to water and standard rodent chow (Formulab Diet 5008C33, Richmond, Indiana) prior to experiments. Mice between the ages of 5–7 months made up the young adult group, and mice between the ages of 24–25 months mice made up the old group. In the first experiment, old mice received either chow or HS diet for 4 weeks. For the second experiment, mice were individually assigned to one of the 2 groups within each cage to undergo surgery for either osmotic pump implantation (Alzet, Cupertino, CA, USA) for ELAM (3 mg/kg bodyweight/day) or saline (control) delivery for two weeks. ELAM was provided by Stealth BioTherapeutics (Newton, MA, USA). One week after pump implantation, mouse cages were randomly selected to remain on standard chow or be provided with HS diet (HS; Bio-Serv, Frenchtown, NJ, USA) (see Table 1 for diet composition). Food intake was recorded per cage and divided by the number of animals in the cage. All animals remained group-housed throughout the duration of the study.

### 2.2. In Vivo Plantarflexor Function

Contractile function of plantarflexor muscles was assesed using the Aurora Scientific 305C servomotor (Aurora, ON, Canada) as previously described [13]. Briefly, each mouse was anesthetized under 2% isofluorane with heat support. The knee was clamped in place and the foot was secured with the ankle at 90° to a foot plate connected to a force transducer. The tibial nerve was stimulated with electrodes. A series of twitches and tetanic contractions were used to optimize voltage (1–5 V). Maximal torque was obtained from a force frequency curve performed by stimulating the muscle at 10, 30, 50, 80, 100, 120, 150, 180, and 200 Hz.

### 2.3. Echocardiography

Transthoracic echocardiography was performed using a Siemens Acuson CV-70 (Munich, Germany) as previously described [7]. Briefly, each mouse was mildly anesthetized under 0.75–2% isofluorane with heat support and heart rate monitoring. Heart rate was maintained in the range of 450–550 beats per minute during the procedure.

### 2.4. Serum Analyses

Insulin levels were measured using ELISA (EMD Milipore, Temecula, CA, USA) according to manufacturer instructions. Blood glucose was assessed with a commercial glucometer.

### 2.5. Mitochondrial Isolation and Respirometry

Mitochondria were isolated from ~100 mg of heart tissue using previously published methods [14]. Freshly isolated mitochondria were injected into a 2 mL chamber of an Oxygraph 2K dual respirometer/fluorometer (Oroboros Instruments, Innsbruck, Austria) that was maintained at 37 °C and stirred gently during substrate and inhibitor titrations. Hydrogen peroxide emission was measured in parallel with oxygen consumption using Amplex Red (50 uM) and HRP (0.1 U/mL) (Invitrogen, Waltham, MA, USA). Substrates to induce leak respiration included pyruvate (5 mM), glutamate (10 mM), and malate (2 mM), followed by state 3 respiration under complex I substrates with ADP (2.5 mM) and then by fatty acid octanoylcarnitine (0.2 mM). Maximum respiration (oxidative phosphorylation; OXPHOS) was induced with the addition of succinate (10 mM) and cytochrome C (10 μM). Next, complex I was inhibited with rotenone (0.5 μM), and mitochondrial respiration was completely inhibited with antimycin A (2.5 μM). N,N,N′,N′-Tetramethyl-p-phenylenediamine dihydrochloride (TMPD; 0.5 mM), ascorbate (2 mM), and potassium cyanide (KCN; 1 mM) were then added to assess Complex IV capacity.

### 2.6. Histology

Hearts were fixed in 10% formalin and were cross-sectionally cut close to the apex of the heart to be paraffin embedded by the University of Washington Histology Core. Sections were then stained with hematoxylin and eosin using standard staining methods, and with picrosirius red to visualize collagen. Cardiomyocyte diameter and the proportion of red stained collagen to whole stained area were measured using Image J software version 1.52a (NIH, Bethesda, MD, USA).

### 2.7. Gene Expression

Frozen longitudinal halves of heart tissue were pulverized with a liquid nitrogen-cooled mortar and pestle. About 30 milligrams of tissue was further homogenized with the QIAshredder followed by RNA isolation using the RNeasy Fibrous Tissue Mini Kit (Qiagen, Germantown, MD, USA). 12 ng of RNA was then used for RT-PCR (7500 Real-Time PCR Systems) using the Verso-1-step RT-qPCR mix (Invitrogen). Primers for Myh7 (Mm00600555_m1), GAPDH (Mm03302249_g1), Pdk4 (Mm01166879_m1), Cpt1 (Mm00487191_g1), IL1b (Mm0043228_m1), TNF (Mm00443258_m1) were used for qPCR. The delta-delta threshold cycle (2^−ΔΔCt^) was calculated to determine the relative difference in expression for each gene of interest from the young-chow group, unless noted otherwise, using GAPDH as the housekeeping gene.

### 2.8. Western Blotting

Tissue was homogenized in CellLytic buffer (Sigma). Protein was quantified by Bradford assay. Equal amounts of protein from cardiac homogenate or isolated mitochondria were loaded into a Tris-Glycine 4–20% gradient gel (Biorad). Antibodies included total OXPHOS (Abcam: ab10413), anti-RAGE (Abcam; ab37647), O-GlcNAc (Cell Signal; CTD110.6), and 4-HNE (Alpha Diagnostics; HNE12S).

### 2.9. Statistical Analyses

Data presentation and analyses were performed using GraphPad Prism software version 9 (San Diego, CA, USA). Data are expressed as mean ± standard deviation. Student’s two-tailed *t*-tests were used to compare two groups; one-way ANOVA tests were performed to compare three groups; and two-way ANOVA tests were employed to determine the effect of treatment and age. Following ANOVA tests, a Dunnet post hoc test was used to determine differences to age-matched chow-fed controls. *p*-values under 0.05 were considered significant.

## 3. Results

### 3.1. Old Mice Consuming a HS Diet for More Than 1 Week Became Frail and Developed Exacerbated Cardiac Hypertrophy

**Figure 1:**
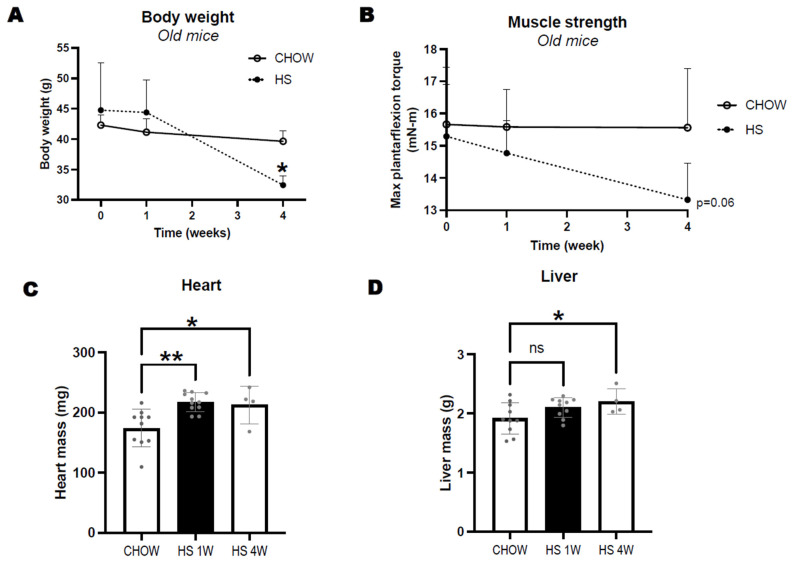
Changes in body weight (**A**) and maximal isometric torque of plantarflexors (**B**) in 24-month-old CB6F1 mice consuming either standard chow or high sucrose (HS) diet for 1 or 4 weeks. Differences in heart (**C**) and liver (**D**) mass between groups. Data expressed as mean ± SD. Significant differences to age-matched chow are indicated by * *p* < 0.05, ** *p* < 0.01, non–significant effects by "ns".

We provided 24-month old male CB6F1 mice with HS diet where all carbohydrate-derived calories came from sucrose (60%kcal). Mice were weight stable until after 1 week on HS diet, but by week 4 they lost 15% of their body weight and 18.5% of muscle torque (*p* = 0.06 vs. chow) (Figure 1A,B), which led us to discontinue the long-term HS diet. A group of mice was also euthanized after 1 week of HS diet when body weight was similar to age-matched controls. After 1 week of HS diet, we found that hearts were significantly larger than chow-fed, age-matched mice (Figure 1C). This hypertrophy persisted, but did not increase after 4 weeks. Liver weight was also larger following 4 weeks of HS diet (Figure 1D).

### 3.2. HS Diet for 1 Week Does Not Lead to Obesity or Hyperinsulinemia in Either Young or Old Mice

**Figure 2:**
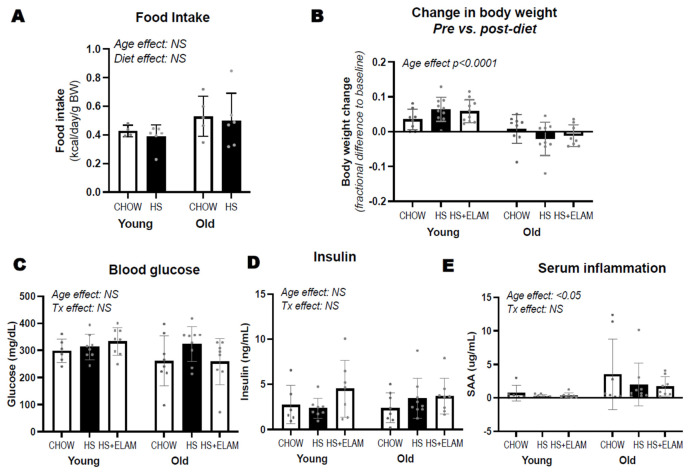
Differences in food intake (**A**), changes in body weight (**B**), and non-fasted serum glucose, insulin, and serum amyloid A (SAA) (**C**–**E**) between young adult and old adult male CB6F1 mice consuming regular chow or HS diet, treated with or without elamipretide (ELAM). Food intake was recorded per cage housing both saline and ELAM–treated groups. Two-way ANOVA was used to detect differences between age and treatment (Tx) groups. Significant effects are denoted by *p*–value and non–significant effects by “NS”. Data expressed as mean ± SD.

In a second experiment, we compared the effect of 1 week of HS diet on food intake and body weight of young and old mice treated with or without ELAM. Despite a tendency for old mice to consume more calories per body weight than young mice (age effect *p* = 0.09; Figure 2A), young mice increased their weight and old mice did not. Mice on HS diet did not have a higher caloric intake than those on chow. However, HS diet promoted weight gain in young mice but not in old mice (Table 2, Figure 2B). We did not detect differences in non-fasting glucose or serum insulin levels between groups (Figure 2C,D), though old mice tended to have lower glucose levels (*p* = 0.1). We also detected an increase in inflammatory marker serum amyloid A (SAA) in old mice, but HS did not increase SAA in either age group (Figure 2E).

### 3.3. HS Diet for 1 Week Leads to Cardiac Hypertrophy with Preserved Function Specifically in Old Mice, an Effect Prevented by Elamepretide

**Figure 3:**
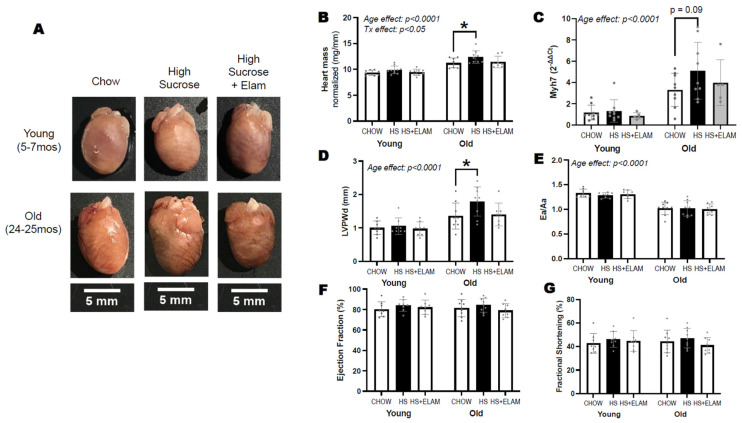
Representative images of dissected hearts from each group on the same scale (**A**). Heart mass normalized to tibial length (**B**). Gene expression of hypertrophy marker myosin heavy chain 7 (Myh7) is expressed relative to young–chow (**C**). In vivo measures of cardiac parameters by echocardiography include left ventricular posterior wall dimension (**D**). Ea/Aa ratio as indicator of diastolic function (**E**), and ejection fraction and fractional shortening as indicators of systolic function (**F**,**G**). Significant differences between old and young are indicated by age effect; differences between old–chow and old–HS are indicated with smaller brackets containing *p*–values if slightly above *p* > 0.05 or asterisks if significant, *p* < 0.05. Data expressed as mean ± SD.

To determine whether there was an age-dependent effect of a short-term HS diet on cardiac tissue, we compared heart weight, gene expression of hypertrophy marker Myh7, and left ventricular posterior wall dimension (LVPWd) by in vivo echocardiography in young and old mice fed HS diet for 1 week. A group of mice received ELAM for two weeks (one week prior and the week during HS diet) to detect whether diet-induced effects could be prevented by mitochondrial therapy treatment. As expected, hearts from old mice were larger, as evidenced by heart mass, Myh7 expression, and echocardiography (Figure 3A–D). In experiment 1, the hearts of old-HS mice were significantly larger compared to age-matched groups, but hearts from the ELAM-treated, old mice on HS did not differ from those old mice on chow group. Furthermore, we did not detect differences in heart mass in young-HS mice relative to age-matched groups. To determine whether HS diet would exacerbate cardiac dysfunction, we measured Ea/Aa ratio as an indicator of diastolic function, and fractional shortening and ejection fraction as indicators of systolic function. We found age-related decline in diastolic function, but no differences in systolic function (Figure 3E–G). We also did not detect differences in cardiac function with HS in the presence or absence of 2-week ELAM treatment.

### 3.4. Isolated Mitochondria from HS-Fed Mice Did Not Exhibit Significant Differences in Function or Protein Content

**Figure 4:**
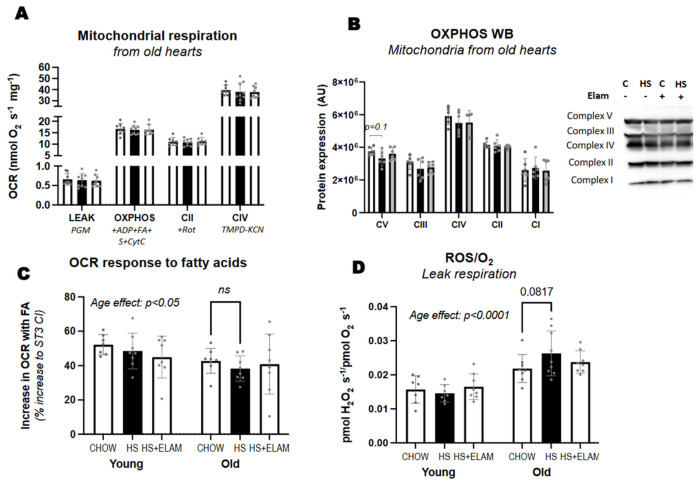
Mitochondrial respiration and hydrogen peroxide production. Mitochondrial respiration using Oroboros O2K (**A**,**C**) and protein content of electron transport chain complexes assessed by immuno blot (**B**) were measured in isolated mitochondria from freshly isolated hearts. The rise in respiration with the addition of fatty acid (FA) octanoylcarnitine in the presence of complex I substrates and ADP was quantified for each age and treatment group (**C**). The ratio of reactive oxygen species (ROS) to oxygen being consumed by isolated mitochondria (**D**). Significant differences between old and young are indicated by age effect; differences between old-chow and old-HS are indicated with smaller brackets containing *p*–values between 0.05–0.1 or asterisks if significant, *p* < 0.05, or NS if *p*–value is above 0.1. Data expressed as mean ± SD.

Mitochondrial function needs to be adequate to meet the high metabolic demand of the heart. To determine whether mitochondrial function is affected by HS diet, we measured oxygen consumption rates using high-resolution respirometry of isolated mitochondria in the presence of a variety of substrates. We found no effect of HS diet with or without ELAM on leak respiration in the presence of complex I substrates; maximum oxidative phosphorylation (OXPHOS) measured after adding saturating ADP in the presence of pyruvate, glutamate, malate (PGM), octanoylcarnitine (FA), succinate (S), and cytochrome C; or complex II (+Rotenone) or IV activity (TMPD-KCN) (Figure 4A). We also did not detect differences in protein content of OXPHOS complexes (Figure 4B). We then compared the rise in respiration when adding octanoylcarnitine following the addition of ADP + complex I substrates, and we found an age-related decrease in fatty acid-induced respiration (Figure 4C), indicative of reduced capacity for fatty acid oxidation. However, HS diet with or without ELAM did not aggravate this age-associated effect. Since previous studies have shown that antioxidants can prevent HS-induced cardiac hypertrophy, we measured hydrogen peroxide production by isolated mitochondria in the leak state relative to oxygen consumption. As expected, we found an age-associated increase in ROS/O_2_ in old mice, but no detectable effect of HS or ELAM (Figure 4D).

### 3.5. Old Hearts Have Increased Inflammation and Fibrosis, as Evidenced by Gene Expression and Histology, That Was Not Significantly Affected by HS Diet with or without ELAM

**Figure 5:**
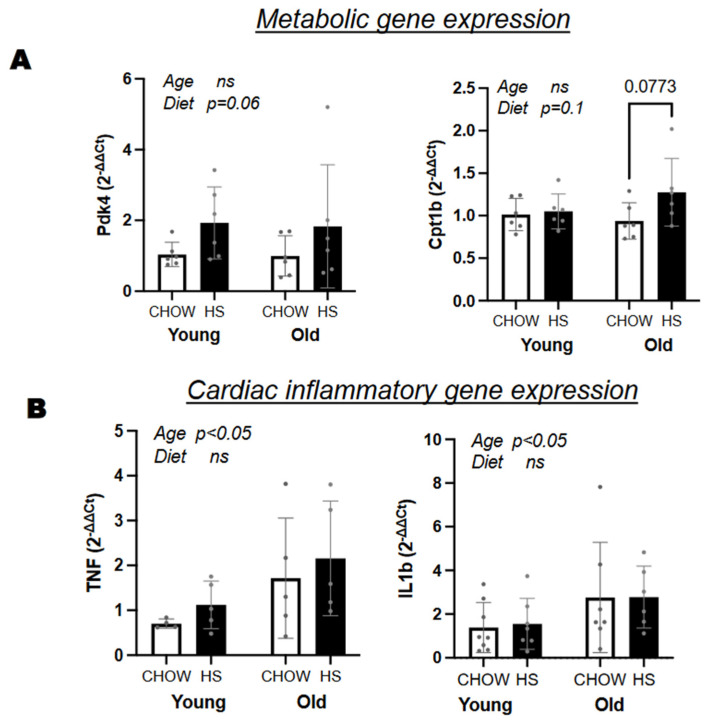
Gene expression and histology of heart tissue. (**A**) Differences in metabolic and (**B**) inflammatory genes between diet and age groups; (**B**). Age and diet effects are indicated with “ns” if above 0.1, with *p*-value if between 0.05–0.1, and significant if below 0.05. Data expressed as mean ± SD.

The adult heart obtains ATP mainly through fatty acid oxidation. During pathological cardiac hypertrophy there is a shift in the metabolic profile that leads to a reduction in fatty acid and glucose oxidation and an increased reliance in glycolysis. We wanted to determine if there were changes in the expression of genes responsible for the selection of substrates. Pyruvate dehydrogenase regulates mitochondrial metabolism by promoting the switch to glucose oxidation when activated or to fatty oxidation when inhibited by its kinases, such as pyruvate dehydrogenase kinase 4 (PDK4). Carnitine-palmitoylacyltransferase-1 (CPT1), the rate-limiting enzyme of fatty acid oxidation in the heart, is located on the outer surface of mitochondria where it esterifies fatty acids. We found no differences in PDK4 or CPT1b expression between the two age groups. HS diet increased PDK4 expression, but this effect did not reach statistical significance (Figure 5A). CPT1b gene expression also increased in the aged hearts with HS diet, but this effect also did not reach statistical significance (Figure 5A). No differences were detected in the HS + ELAM group (Figure S2).

We also tested whether the elevated exposure HS and age-related redox stress in aged mice consuming HS would result in increased post translational modifications associated with redox stress (4-hydroxynonenal) and exposure to sugars O-linked N-acetylglucosamine (O-Glc-NAc) and receptor for advanced glycation end products (RAGE). We did not detect differences by HS or ELAM in any of these markers (Figure S1).

We suspected that increased heart weight with HS diet could be reflective of an increase in inflammation. However, we were unable to detect differences in gene expression of inflammatory genes IL1b and TNF by diet (Figure 5B) or by HS + ELAM (Figure S2). However, aged hearts had a higher expression of these inflammatory genes compared to young hearts. A similar trend was seen for circulating levels of serum amyloid A (Figure 2E).

**Figure 6:**
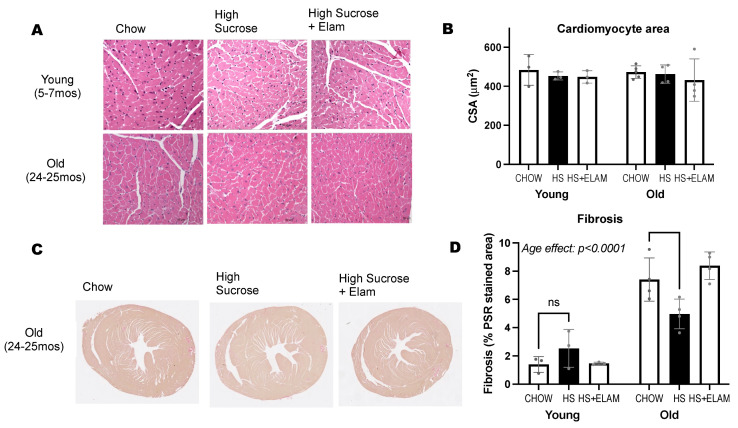
Histological analysis of heart tissue. Sections of heart apex stained with H&E (**A**) and Picrosirius Red (**C**) to measure cardiomyocyte size (**B**) and collagen content (**D**). Significant differences between old and young are indicated by age effect *p* < 0.05. Differenced between old–chow and old–HS are indicated with a smaller bracket containing the *p*–value or asterisk if <0.05. Data expressed as mean ± SD.

A histological analysis of the hearts was conducted to measure cardiomyocyte size and fibrosis (Figure 6). Despite larger heart weights and left ventricular wall thickness, we were unable to detect differences in cardiomyocyte size between the two age groups and treatment groups. We found increased levels of collagen in aged hearts. Unexpectedly, hearts from the old HS-fed mice had significantly reduced fibrosis compared to age-matched controls.

## 4. Discussion

Here, we report that hearts from old mice are more susceptible to nutritional stress by a high sugar diet compared to hearts from young mice. Hypertrophy is a common feature of cardiac pathology in aged mice. We show that this process is accelerated at as early as 1 week of high sucrose (HS) consumption through mild yet significant differences in heart mass in two separate experiments: left ventricular wall dimension and hypertrophic gene expression. Cardiac hypertrophy induced by HS diet also persisted at week 4, even after the mice lost 15% of their body weight. Old mice treated with the mitochondrial-targeted drug, ELAM, were protected from the HS diet-induced effect. To our knowledge, this is the first published investigation of age-differences in cardiac response to a short-term HS diet.

Cardiac hypertrophy increases the risk of developing heart failure and malignant arrhythmias [15,16]. Aging is a major risk factor for heart failure almost doubling every 10 years [16]. In our study, we detected common features of normal cardiac aging, including left ventricular hypertrophy and fibrosis. In addition, aging is associated with low grade chronic inflammation, which was also detected in our study with increased serum amyloid A and inflammatory gene expression in the heart. Previous studies have shown that low grade inflammation increases susceptibility to nutritional stressors. For instance, mice lacking NLRP3 were protected from developing cardiac hypertrophy induced by HS diet [17]. HS diets, particularly high-fructose diets, also elevated plasma lipopolysaccharide levels and TNF-alpha concentrations in young and adult rats after only 2 weeks despite no differences in body weight [18]. However, while aged mice had increased inflammation, HS diet did not appear to exacerbate it after 1 week in this present study. In addition, protection from cardiac hypertrophy by ELAM was not associated with reduced serum amyloid A.

Mitochondria are essential to provide energy to the heart, and reactive oxygen species (ROS) result as byproducts of mitochondrial respiration that can be quenched by antioxidant systems. Previous studies have shown mitochondrial-targeted antioxidant SkQ1 or systemic antioxidant naringin in combination with high sugar feeding protect the heart from developing hypertrophy [9,10]. There are also improvements in mitochondrial bioenergetics, redox stress, and cardiac function in the aging heart following 8 weeks of ELAM treatment [6,7]. In this present study, we found that isolated mitochondria from aged hearts produce a greater amount of ROS, similar to previous findings [6,19]; however, we did not detect differences in ROS production, lipid peroxidation, or mitochondrial respiration in isolated mitochondria from hearts of aged animals treated with HS or HS + ELAM. Our findings suggest that that ELAM may be acting systemically to reduce the pathological signaling of HS consumption. Our group and others have also found that improvements in mitochondria and tissue function by ELAM extend beyond the myocardium, including the kidney, retina, and skeletal muscle [20,21,22]. While the focus of this study was on cardiac tissue, a more comprehensive analysis of other metabolic tissues (e.g., liver) may provide a clear answer as to how HS induces cardiac hypertrophy and how ELAM is protective against it. Here, we show that the benefits of ELAM treatment extend to prevention of cardiac remodeling induced by HS diet.

It is possible that the age-related sensitivity to HS diet in the heart resulted from age-associated differences in tissues that were not examined in this study, such as the liver, intestine, kidney, or adipose tissue. In fact, we found an age-dependent effect on weight gain by HS diet, as the HS diet increased body weight of young mice but decreased body weight of old mice. Therefore, age-related differences in the response of adipose tissue to a short-term HS diet may have protected young mice from developing cardiac hypertrophy, and HS diet may have exposed a reduced capacity by aged adipose tissue to induce de novo lipogenesis or store sucrose-derived triglycerides [23]. De novo lipogenesis is also activated in the liver when consumption of sugar is high [24], and while liver steatosis was not analyzed in this study, the differences in liver weight predominantly explained age at the 1-week time point, and was significantly greater with HS at the 4 week timepoint, may have been reflective of increased lipid deposition. Fructose, which makes 50% of sucrose, is predominantly metabolized by the intestine and liver, and has a strong association with metabolic dysfunction [3,25,26,27]. Hepatic fructose metabolism can lead to de novo lipogenesis and uric acid production [26,28,29] that are associated with left ventricular hypertrophy in people with hypertension, chronic kidney disease, and congestive heart failure [30,31].

Poor nutrition, particularly the Western diet, is often linked to heart disease. Here, we show that high sucrose without excess fat or cholesterol content normally used in Western diets, is enough to stress aged cardiac tissue in as little as one week, in the absence of weight gain or significant changes in blood glucose and insulin. It is possible that we were unable to detect differences as the mice were not fasted prior to blood collection. Nevertheless, previous studies have shown that HS diets can promote pathological cardiac remodeling, endothelial dysfunction, and hepatic steatosis in the absence of insulin resistance, increased blood pressure, or weight gain [11,32,33]. Therefore, changes in cardiac size as a result of an HS diet, may be difficult to detect in humans due to the absence of common metabolic parameters used clinically to assess metabolic health (e.g., blood glucose, body fat, insulin sensitivity).

Future work should test the effect of sugar consumption in aged female mice. Females exhibited a greater preference for sugar by consuming 50% more sucrose-sweetened water than males [34]. The withdrawal of circulating estrogen following menopause also increases the risk for cardiovascular events in women [35]. Ovarian hormones can be protective by promoting the expression of antioxidant enzymes, such as glutathione peroxidase 1 (Gpx1), in the liver [36]. Therefore, age-related differences in susceptibility to nutritional stress may be more pronounced in females than in males because of the reduction in ovarian hormone levels with menopause. Future studies should also address the impact of chronic sugar consumption in the context of aging. A limitation of this present study was that mice became too frail when exposed to HS diet longer than 1 week. The diet used in this study represented an extreme diet stressor as a first step in detecting an age-related difference in response. Diets where sugar-derived energy is reduced to >10–25% may be more amenable to older mice, while also being translatable to humans. Despite this limitation, the high content of sucrose allowed us to detect striking age-related differences. Our work supports previous work performed on younger rodents where sugar consumption aggravates the heart and highlights the vulnerability of the aging heart to stress by dietary sucrose.

## Figures and Tables

**Table 1:** Diet composition.

Diet		Chow (Picolab 5008C33)	High Sucrose (HS) (Bioserv Custom)
Composition by weight	Protein (%)	~23.6	20.5
	Fat (%)	~6.7–8.1	7.0
	Carbohydrate (%)	~50.3	65.8
	Sucrose (%)	~3.23	65.8
Composition by calories	Protein (%kcal)	27	20
	Fat (%kcal)	17	15
	Carbohydrate (%kcal)	57	64
	Sucrose (%kcal)	~3.6	64
	Physiological fuel value (kcal/g)	3.56	4.08

**Table 2:** Mouse and tissue weights.

Variable	Young Chow	Young HS	Young HS + ELAM	Old Chow	Old HS	Old HS + ELAM	Age-Effect *p*-Value	Treatment Effect *p*-Value
Sample size (*n*)	8	10	10	9	9	8		
Body weight pre-diet (g)	37.06 ± 2.39	36.98 ± 2.36	35.96 ± 4.32	41.14 ± 3.96	43.98 ± 5.09	40.64 ± 2.97	<0.0001	-
Body weight post-diet (g)	38.34 ± 2.32	39.32 ± 2.29 ^$^	38.0 ±4.11 ^$^	41.56 ± 4.96	42.69 ± 3.66	40.13 ± 2.39	<0.01	0.25
Heart (mg)	174.03 ± 12.55	184.32 ±14.34	179.1 ± 18.52	216.03 ± 18.52	237.76 ± 21.9 *	218.91 ± 18.71	<0.0001	<0.05
Liver (g)	1.67 ± 0.09	1.77 ± 0.25	1.85 ± 0.25	2.14 ± 0.34	2.41 ± 0.38	2.18 ± 0.26	<0.0001	0.16
Gastrocnemius (mg)	188.23 ± 9.76	192.89 ± 13.13	185.83 ± 20.09	176.97 ± 19.9	179.89 ±13.06	174.63 ± 17.28	<0.0001	0.5

Values are expressed as mean ± SD. ^$^ *p* < 0.05 paired *t*-test vs. pre-diet. * *p* < 0.05 compared to age-matched chow Dunnett’s multiple comparisons test.

## Data Availability

Data is available upon request to corresponding authors.

## Supplementary Materials

The following supporting information can be downloaded at: https://www.mdpi.com/article/10.3390/nu14214645/s1, Figure S1: Western blot of 4-HNE, RAGE, and O-GlcNAC; Figure S2: Expression of inflammatory and metabolic genes in aged heart tissue with and without ELAM.
