# Supplementary material for: Aging Increases Susceptibility to Develop Cardiac Hypertrophy following High Sugar Consumption
Source: Nutrients. 2022 Nov 3;14(21):4645. doi: 10.3390/nu14214645 (PMC9655368; doi:10.3390/nu14214645)
Supplement: Supplementary file 1 [file nutrients-14-04645-s001.zip › nutrients-1926984-supplementary.pdf]

AGING INCREASES SUCEPTIBILITY TO DEVELOP CARDIAC HYPERTOPHY FOLLOWING HIGH SUGAR CONSUMPTION

Ana P. Valencia, Jeremy A. Whitson, Shari Wang, Leon Nguyen, Laura J. den Hartigh, Peter S. Rabinovitch, and David Marcinek.

Supplementary material

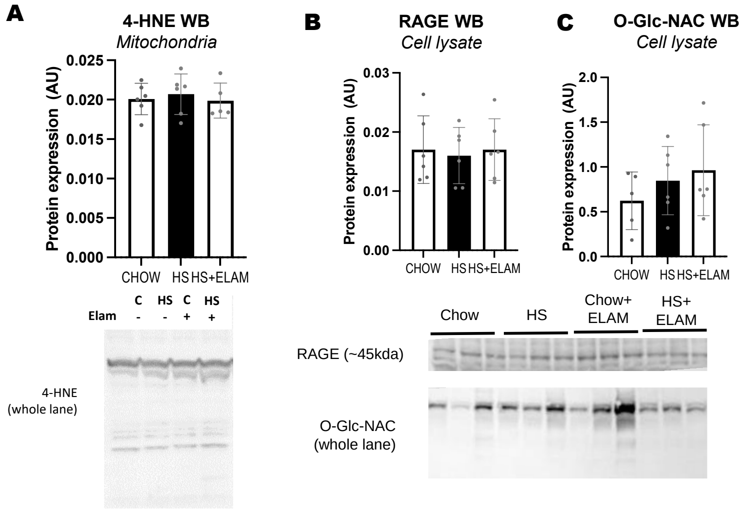

Figure S1: Western blot of 4-HNE, RAGE, and O-GlcNAC on mitochondria or cell lysate as indicated, from 24 month old mice consuming chow, HS, HS+Elam. Blots also show a parallel chow-fed ELAM group that did not undergo further analysis for the purpose of this study. Data expressed as mean +/- SD.

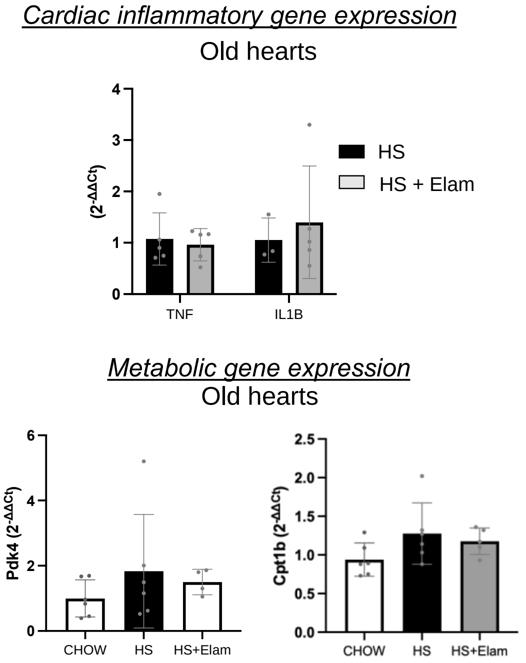

Figure S2: Expression of inflammatory and metabolic genes in aged heart tissue with and without ELAM. Inflammatory genes are shown relative to age-matched HS group, and metabolic genes relative to age-matched chow group. Data expressed as mean +/- SD.
